# Optimal Feeding Rates for Growth Performance, Nutrient Retention, and Heat Shock Protein 70 Expression in Fingerling Yellow Perch (*Perca flavescens*)

**DOI:** 10.3390/ani15101465

**Published:** 2025-05-19

**Authors:** Shao-Wei Zhai, Xing Lu, Song Yang, Fred P. Binkowski, Dong-Fang Deng

**Affiliations:** 1 School of Freshwater Sciences, University of Wisconsin-Milwaukee, Milwaukee, WI 53204, USA; zhaisw@jmu.edu.cn (S.-W.Z.); luxing@gdim.cn (X.L.); ysys210@hotmail.com (S.Y.); sturgeon@uwm.edu (F.P.B.); 2 Fisheries College, Jimei University, Xiamen 361021, China; 3 Institute of Microbiology, Guangdong Academy of Sciences, Guangzhou 510070, China; 4 College of Animal Science and Technology, Sichuan Agricultural University, Chengdu 611130, China

**Keywords:** feeding rate, growth, HSP70, nutrient retention, yellow perch

## Abstract

Yellow perch (*Perca flavescens*), a native species of the Great Lakes, has experienced a significant decline in wild harvest since the 1990s, leading to an increased reliance on aquaculture. This study aimed to optimize feeding rate for yellow perch by evaluating the effects of various feeding rates (1.5%, 3.0%, 4.5%, 6.0%, and 7.5% body weight (BW)/day) on growth, nutrient retention, and heat shock protein 70 expression in perch fingerlings (initial BW: 1.73 ± 0.11 g) over a four-week period. Following the feeding trial, an acute heat shock was induced by raising the water temperature from 23 °C to 31 °C, followed by an 18 h recovery period. Results indicated that both growth rate and whole fish lipid content increased with higher feeding rates (*p* < 0.05), while nutrient retention decreased. Growth and energy retention did not significantly improve beyond 4.5%, with maximum energy retention observed at 3.9% BW/day. Heat shock protein 70 expression was highest in liver tissue at a feeding rate of 6.2% BW/day, indicating that higher feeding levels may enhance thermal stress response. These results indicate that a feeding rate of 4.5% BW/day supports optimal growth, while 6.2% BW/day may be necessary to improve heat shock tolerance. The study provides practical insights for refining feeding strategies in yellow perch aquaculture.

## Simple Summary

Yellow perch are ecologically and economically important fish in the Great Lakes region. This study aimed to optimize feeding practices for yellow perch in aquaculture, with the goal of enhancing both growths, understanding the impact of nutrition status on heat shock tolerance. Specifically, we sought to determine the ideal balance of feed quantity for promoting growth, improving nutrient utilization, and increasing the fish’s ability to withstand acute temperature changes. Our results indicated that a feeding rate of 4.5% BW represents an optimal balance between FCR and growth rate. A rate of 6.2% BW is recommended to achieve maximum growth and heat shock protein 70 expression. A feeding rate of 3.0% BW may be suitable for maintaining a low FCR without severely compromising growth. Inadequate feeding not only reduced growth, but also negatively impacted lipid storage and might reduce heat stress tolerance capacity in perch fingerlings. These findings provide valuable insights into yellow perch farming practices and nutritional research of this fish.

## 1. Introduction

Yellow perch (*Perca flavescens*) is a culturally significant freshwater fish, often associated with the “Friday Fish Fry” tradition in the Midwestern United States [1]. In the Great Lakes, the commercial yellow perch fishery exceeded 33 million pounds annually during the 1950s and 1960s. However, commercial fishery had declined to 11 to 18 million pounds per year by 1990 [1,2,3]. Despite this decline, market demand for yellow perch remains strong with fillet prices reaching USD 20 to 30 per pound. This sustained demand has driven the growth of commercial perch aquaculture.

The industry is composed of two primary sectors: the production of fingerlings for personal and community recreational ponds or lakes, and the production of market-size fish for direct human consumption. Compared to many other aquaculture species, yellow perch have a slower growth rate; however, their market size—typically 115 to 150 g per whole fish—is smaller than that of most other farmed food fish [3]. One significant challenge in yellow perch aquaculture is cost-effective production of high-quality fingerlings. Fingerlings have high metabolic rates and fast early growth, making them especially sensitive to nutrition deficiency if not provided with appropriate feeding [4]. Establishing optimal feeding practices is therefore crucial for successful fish hatchery. Inadequate feeding can impair health and slow growth [4,5,6], while overfeeding increases production cost, degrades water quality, and can overload the digestive system, reducing feed efficiency [7,8,9,10].

To date, no study has comprehensively evaluated optimal feeding strategies for yellow perch fingerlings. Furthermore, feeding rates not only affect growth performance but can also change the nutritional status of fish and ability to respond to environmental stress. One well-established biomarker for assessing stress is heat shock protein 70 HSP70 [11,12,13,14,15], a highly conserved protein involved in stabilizing cellular proteins to maintain cell integrity [16,17]. HSP70 expression has been shown to vary in response to nutritional status in fish, including starvation and feeding rate changes, suggesting that HSP70 could serve as a potential cellular biomarker for assessing fish feeding [12,18].

In this study, we aim to evaluate the growth performance and HSP70 expression of yellow perch fingerlings in response to different feeding rates and acute heat shock. The goal is to gain a better understanding of the relationship between feeding management, growth, nutritional status, and heat shock response in yellow perch fingerlings. The findings will provide important insights to inform cost-effective management yellow perch aquaculture.

## 2. Materials and Methods

### 2.1. Fish Source and Maintenance

Yellow perch larvae were produced from broodstock cultured at the School of Freshwater Sciences, University of Wisconsin-Milwaukee, Milwaukee, WI, USA, and were raised until used for the current feeding trial. All procedures were conducted in accordance with the animal protocol approved by the Campus Animal Care and Use Committee (Protocol Number 15-16#46).

Fish were allowed to acclimatize for two weeks in the same tanks (1.0 m diameter, 71 cm height, partially filled with 270 L water per tank) used for the feeding trial. They were maintained according to the protocols followed during the 4-week feeding trials. All tanks were continuously aerated and received flow-through water at a flow rate of 3.3 L/min, with 65 fish per tank. The indoor culture system operated using dechlorinated municipal flow-through water. Temperature was controlled by adjusting the flow rates of chilled, cold, and hot water into a central sump, allowing the system to maintain a consistent, targeted water temperature. The fish were fed four times daily to apparent satiation with a commercial feed (Zeigler Bros., Inc. 400 Gardners Station Road, Gardners, PA, USA; moisture 6.5%, protein 55.5%, crude fat 15.4%, and ash 11.0%). The daily feed was divided into four equal portions and hand-fed at 08:30, 11:00, 13:30, and 16:00 h.

Water quality was monitored daily for temperature and dissolved oxygen, and weekly for ammonia and pH. During both the acclimatization period and the 4-week feeding trial, water temperature was maintained at 22 ± 0.5 °C, pH at 8.0 ± 0.1, ammonia nitrogen levels were kept below 0.01 mg/L, and dissolved oxygen remained above 7 mg/L. The diurnal light/dark cycle was set to 12 h of light and 12 h of dark. All tanks were cleaned daily by siphoning to remove waste before feeding.

At the end of the two-week acclimatization period, uniformly sized fish (body weight: 1.73 ± 0.053 g, *n* = 15) were selected and randomly distributed into 15 circular fiberglass tanks, with 50 fish per tank. A group weight was recorded for each tank to ensure uniformity, and the coefficient of variation (CV) across tanks was maintained below 0.10 to minimize variability in initial fish size among treatments. The fish were fed at five different feeding rates: 1.5%, 3.0%, 4.5%, 6.0%, and 7.5% body weight (BW)/day, using the commercial diet described above, with three tanks for each feeding treatment. The feeding trial lasted for 4 weeks. The fish in each tank were weighed every 2 weeks, and the daily feed was adjusted based on the new body weight. The same animal husbandry management practices and water quality conditions were maintained throughout the acclimatization and feeding periods.

### 2.2. Sample and Data Collection

At the start of the feeding trial, three initial samples were collected from the stock tanks, with each sample consisting of 30 fish. The fish were euthanized using an overdose of MS-222 (200 mg/L; Argent, Redmond, WA, USA), blotted dry, weighed as a group, and stored at −80 °C. These samples were used for proximate composition analysis.

At the start, the end of the 2nd week and the 4th week of feeding, the total biomass of each tank was measured after a 20 h period of feed deprivation. Growth rate, survival, total feed fed, and feed conversion ratio were measured. At the end of the 4-week feeding trial, eight fish from each tank were euthanized using MS-222 and measured for body weight and body length to calculate the condition factor (CF). The fish samples were pooled by tank and stored at −80 °C for proximate composition analysis. Additionally, six fish from each tank were dissected to determine liver weight and calculate the hepatosomatic index (HSI). Liver and white muscle tissues were frozen in liquid nitrogen and stored at −80 °C until used for analysis for the basal level of heat shock protein 70 (HSP70) expression.

Tissue collection for heat shock stress: Twelve fish from each tank were placed into two cages, with six fish per cage, which were then submerged in an 8-foot tank running with flow-through water at 23 °C. The water temperature gradually increased to 31 °C at a rate of 1 °C per 15 min using the protocol described above for water temperature control. Fish from the first cage were euthanized when reaching the target temperature, and liver and white muscle tissues were collected for HSP70 analysis. Fish from the second cage were transferred into a recovery system running with flow-through water at 3.3 L/min at 23 °C for 18 h before liver and white muscle tissues were collected for HSP70 analysis.

### 2.3. Sample Analysis

Proximate composition analysis of the test diet and fish samples was conducted following the methods by AOAC [19]. Moisture content was determined by drying the samples in an oven at 105 °C for 12 h or until a constant weight was achieved (AOAC 950.46). Total nitrogen levels were measured using an elemental combustion system (ECS 4010 nitrogen/protein analyzer, Costech Analytical Technologies Inc., Valencia, CA, USA; AOAC 990.03). Crude protein content was calculated by multiplying total nitrogen by 6.25. Crude lipid content was determined by ether extraction (AOAC 2003.05) using a Soxhlet extractor (Soxtec 8000, Foss Analytical, Eden Prairie, MN, USA). Ash content was obtained by combusting the samples in a muffle furnace at 550 °C for 12 h (AOAC 942.05).

The expression of HSP70 in liver and muscle tissues was measured following a similar protocol to that described by Deng et al. [18]. Briefly, frozen tissues were extracted using ice-cold T-PER tissue protein extraction reagent (Thermo Fisher Scientific, Waltham, MA, USA) containing a complete protease inhibitor cocktail (Millipore Sigma, Burlington, MA, USA). Protein concentration in the supernatant was determined using the DC Protein Assay Kit based on the improved Lowry method (Bio-Rad, Hercules, CA, USA). Equal amounts of proteins (25 μg) from each sample supernatant, an HSP70 standard (2 μL), and molecular weight markers (Bio-Rad, Hercules, CA, USA) were loaded onto 10% Tris–HCl precast gels and separated by one-dimensional SDS-PAGE. Molecular weight markers and the HSP70 standard were used to confirm the molecular mass of the protein bands. The separated proteins were electrotransferred onto Immun-Blot Transfer Membranes for subsequent Western blotting analysis and enhanced chemiluminescence, following the protocol by Deng et al. [18]. The primary polyclonal HSP70 antibody was purchased from Enzo Life Sciences (Long Island, NY, USA), and peroxidase-labeled anti-rabbit IgG (Sigma-Aldrich, St. Louis, MO, USA) was used as the secondary antibody to detect the HSP70 probe. Protein bands on the membrane were quantified using the ChemiDoc™ XRS+ System with Image Lab™ Software (Version 6.0.1; Bio-Rad, Hercules, CA, USA). The relative band density of HSP70 was calculated by comparing the band density of each sample to the HSP70 standard on each membrane.

### 2.4. Data Calculation and Statistical Analysis

Weight gain = 100 × (final fish weight, g-initial fish weight, g)/initial fish weight, g.

Specific growth rate (SGR, %/d) = 100 × Ln (final body weight, g/initial body weight, g)/28 days.

Feed conversion ratio (FCR) = (dry feed weight per tank, g)/(total weight gain per tank, g).

Dry feed weight was calculated based on total biomass of each tank x feeding rate x dry matter of feed%.

Protein efficiency ratio (PER) = 100 × weight gain of fish, g/protein fed, g.

Protein retention ratio (PR) = 100 × (final fish body protein, g − initial fish body protein, g)/protein fed, g.

Energy retention ratio (ER) = 100 × (final fish energy − initial fish energy/energy fed. Body and dietary energy were calculated using the following values (kcal g^−1^): crude protein 5.65, lipid 9.40, and NFE 4.23.

Condition factor (CF) = 100 × (BW, g/body length, cm^3^).

Hepatosomatic index (HSI) = 100 × (liver weight, g/final BW, g).

The results are presented as means ± SE of three replicates. Before analysis, data were assessed for normality and homogeneity of variance using the Shapiro–Wilk and Levene’s tests, respectively, using statistical 13.0 software (StatSoft, Tulsa, OK, USA; www.tibco.com/data-science-and-streaming, accessed on 4 April 2025). A one-way analysis of variance (ANOVA) was performed to determine whether there were significant differences in growth performance due to different feedings. Tukey’s HSD post hoc test was used to compare mean values among the different feeding rate groups. The difference in HSP70 expression was subjected to two-way ANOVA to analyze the impact of different feedings, course of stress, and their interaction. Mean values were compared to detect ssignificant difference compared by LSD test. Differences were considered significant at *p* < 0.05. The optimal feeding rate was estimated using second-order polynomial regression [20]. Statistical analysis was performed using StatSoft Statistical 13.0 software (StatSoft, USA www.tibco.com/data-science-and-streaming, accessed on 4 April 2025).

## 3. Results

### 3.1. Growth Performance and Nutrient Retention of Yellow Perch

Growth performance, nutrient retention, and morphology of yellow perch were significantly influenced by feeding rates after four weeks (*p* < 0.05; Table 1). Fish fed between 4.5% and 7.5% body weight (BW) daily showed similar weight gain and specific growth rates (SGR), both significantly higher than those fed at 3% BW/day. The lowest growth occurred in fish fed at 1.5% BW/day. Growth parameters increased with feeding rates up to 4.5% BW/day, with no further improvement beyond this rate. No mortality was observed during the feeding trial.

Feed conversion ratio (FCR) was highest in fish fed 7.5% BW/day, followed by those fed 6% BW/day, which were significantly higher than those fed 1.5% to 4.5% BW/day. Protein efficiency ratio (PER) and protein retention (PR) followed a similar trend, with values for fish fed 1.5% to 4.5% BW/day being significantly higher than for those fed 6% BW/day. Fish fed 7.5% BW/day had the lowest PER and PR. Energy retention (ER) was significantly lower in fish fed 6% to 7.5% BW/day compared to other feeding rates. Condition factor (CF) and hepatosomatic index (HSI) increased with feeding rates (CF: Y = −0.048x^2^ + 0.0657x + 0.8037, R^2^ = 0.792; HSI: Y = −0.0245x^2^ + 0.3328x + 0.4866, R^2^ = 0.8638). The lowest CF and HSI were observed in fish fed at the lowest rate, significantly lower than those fed the highest rate.

Proximate composition, except for protein, significantly correlated with feeding rates (Table 2). Moisture and ash contents decreased, while lipid content increased with higher feeding rates (moisture, Y = 0.0937x^2^ − 1.2072x + 75.331 R^2^ = 0.837; lipid, Y = −0.1302x^2^ + 1.5874x + 3.8165 R^2^ = 0.807; and ash, Y = 0.0229x^2^−0.2852x + 4.0792 R^2^ = 0.932). Fish fed 1.5% BW/day had significantly higher moisture and lower lipid levels compared to those on other feeding regimes. Ash content was significantly higher in fish fed 1.5% and 3.0% BW/day compared to those fed 6% or 7.5% BW/day. Body protein showed a slight increase with feeding rate, but no significant differences were observed across feeding groups.

**Figure 1:**
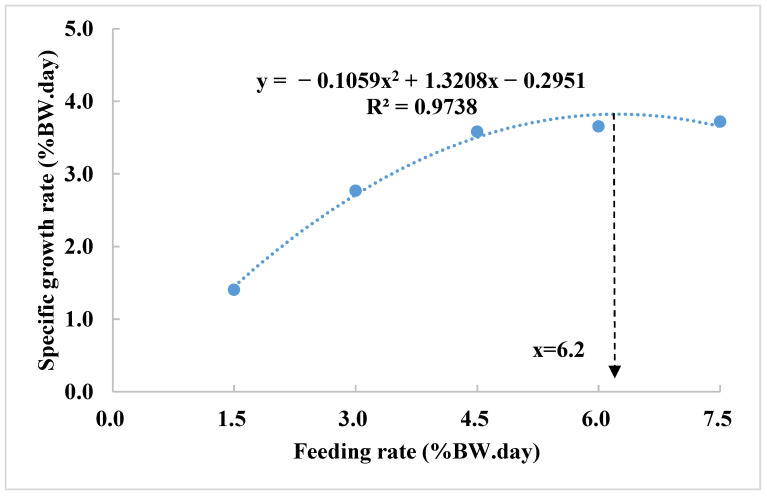
Polynomial regression analysis of the relationship between feeding rate (FR) and specific growth rate (SGR). Data are presented as means based on three replications.

**Figure 2:**
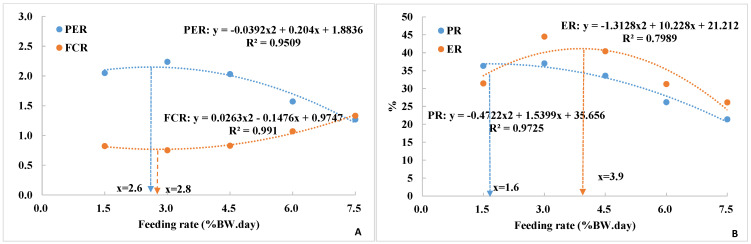
(**A**,**B**) Polynomial regression analysis of the relationship between feeding rate (FR) and protein efficiency ratio (PER), feed conversion ratio (FCR), protein retention (PR), and energy retention (ER) in juvenile yellow perch fed different feeding rates for 4 weeks. Data are presented as means based on three replications.

Polynomial regression analysis identified the optimal feeding rate of 6.2% BW/day for maximum specific growth rate (SGR) (Figure 1). The lowest FCR and highest PER were achieved at 2.8% and 2.6% BW/day, respectively (Figure 2A). The optimal feeding rate to support the highest protein retention (PR) and energy retention (ER) were achieved at 1.6% and 3.9% BW/day, respectively (Figure 2B).

### 3.2. Heat Shock Protein Expression in Liver Tissue

**Figure 3:**
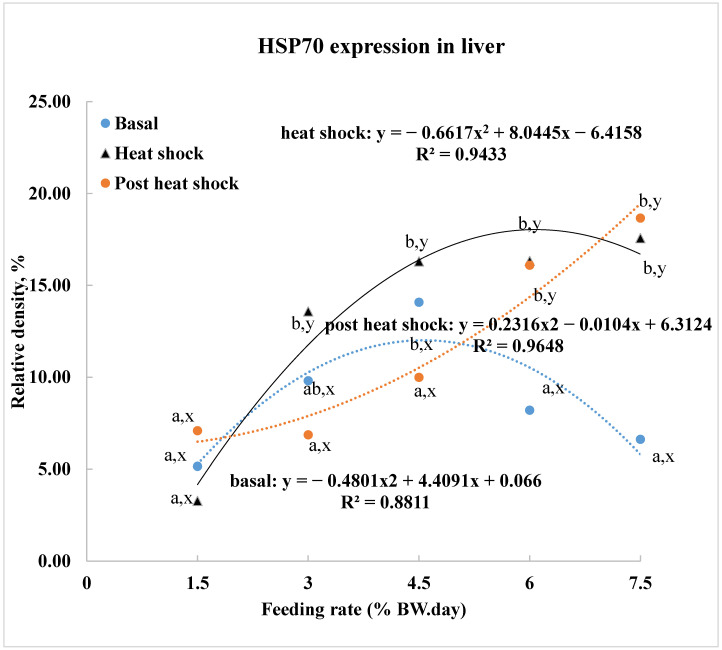
Heat shock protein 70 expression (% relative density to standard) in liver tissues of yellow perch subjected to different feeding rates over four weeks. Data are presented as means based on three replications. Letters a and b denote comparisons across feeding rates; letters x and y indicate comparison between stress conditions. Significant differences were determined at *p* < 0.05.

The basal level of heat shock protein (HSP70) in liver tissue was significantly correlated with feeding rates, as determined by polynomial regression analysis (Figure 3). Fish fed 4.5% BW/day exhibited HSP70 levels similar to those fed 3.0% BW/day but significantly higher than those fed 1.5%, 6.0%, or 7.5% BW/day. Regression analysis indicated that the highest HSP70 expression occurred in fish fed 4.6% BW/day.

Upon exposure to acute heat shock, HSP70 expression increased with feeding rates. The highest expression was estimated in fish fed 6.2% BW/day. However, there was no significant difference in HSP70 expression among fish fed different rates, except for those fed 1.5% BW/day, which showed significantly lower expression. Acute heat shock significantly induced HSP70 expression in all feeding groups, except for the 1.5% BW/day group.

During the post-heat shock period, HSP70 expression remained positively correlated with feeding rates. Fish fed 6.0% or 7.5% BW/day showed significantly higher expression compared to those fed 1.5% to 4.5% BW/day, with no significant differences among the 1.5% to 4.5% BW/day groups. Heat shock and post-heat shock HSP70 levels were similar in fish fed 6.0% to 7.5% BW/day, and both were significantly higher than their basal levels. In contrast, for fish fed 1.5% or 4.5% BW/day, HSP70 expression was similar across basal, heat shock, and post-heat shock periods. Fish fed 3.0% BW/day had similar basal and post-heat shock levels, but their heat shock expression was significantly higher than both basal and post-heat shock levels.

### 3.3. Heat Shock Protein Expression in Muscle Tissue

**Figure 4:**
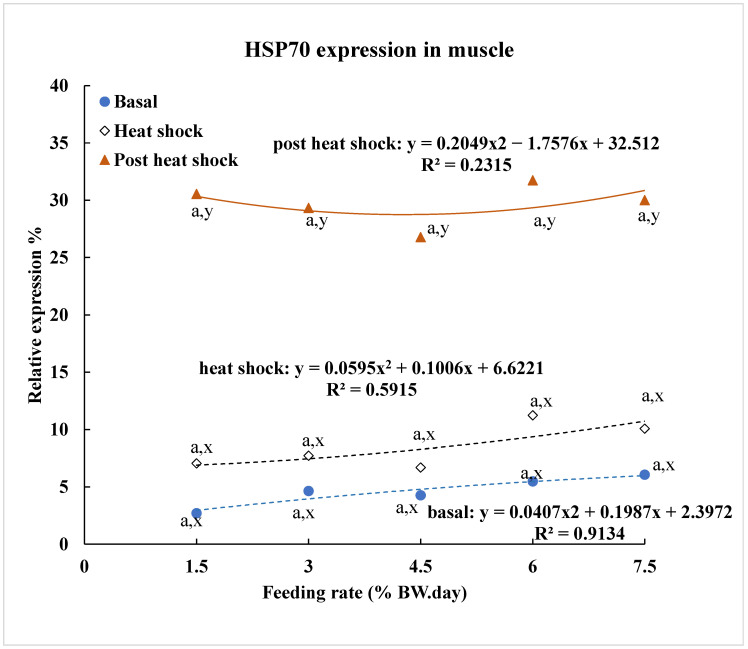
Heat shock protein 70 expression (% relative density to standard) in muscle of yellow perch subjected to different feeding rates over four weeks. Data are presented as means based on three replications. Letters a and b denote comparisons across feeding rates; letters x and y indicate comparison between stress conditions. Significant differences were determined at *p* < 0.05.

Unlike in liver tissue, no significant difference in the basal level of HSP70 expression was observed across various feeding rates in muscle tissue, whether under basal, heat shock, or post-heat shock conditions (Figure 4). However, a significant correlation was found between basal or heat shock levels of HSP70 expression and different feeding rates.

Heat shock induced HSP70 expression in muscle tissue, but no significant difference was observed compared to basal levels. In contrast, post-heat shock HSP70 expression remained significantly higher for all feeding treatments when compared to both basal and heat shock levels.

## 4. Discussion

### 4.1. Growth Performance and Nutrient Retention

The results of this study suggest that a feeding rate of 4.5% BW per day is optimal for growth, nutrient retention, and FCR at this life stage. Both growth and FCR appeared to plateau at 4.5% BW/day, and nutrient retention decreased at higher feeding rates, suggesting that this rate provides the best balance between weight gain and nutrient utilization.

Feeding rates above 4.5% BW/day did not result in significant improvements, indicating diminishing returns beyond this level. FCR ranged from 0.75 to 0.83 across feeding rates from 1.5% to 4.5% BW/day, with no significant differences in FCR, while growth performance improved within this range. However, at a feeding rate of 6.0% BW/day, FCR increased significantly, without a corresponding significant improvement in growth compared to the 4.5% group. This pattern indicates that fish were likely overfed at 6.0% BW/day, reducing feed efficiency while offering no significant growth advantage

Polynomial analysis predicted that a higher feeding rate of 6.2% BW/day could further enhance growth. However, this came at the cost of a 25% increase in FCR (from 0.83 to 1.04), highlighting a trade-off between growth and feed efficiency. Despite this increase, FCR values remained relatively low, ranging from 0.83 to 1.33 across feeding rates of 4.5% to 7.5% BW/day, suggesting that feed utilization remained efficient under these conditions. For smaller fish, like those in this study, a higher feeding rate may be acceptable if the goal is to achieve maximum growth and enhanced physiological response such as increased heat shock protein expression. Conversely, a feeding rate of 3.0% BW/day may serve as a practical compromise, maintaining a low FCR without severely compromising growth. At the lowest feeding rate of 1.5% BW/day, no weight loss or mortality was observed, suggesting that this level met or exceeded the maintenance requirements for yellow perch over the duration of the trial.

Therefore, 4.5% BW/day is recommended as the optimal feeding rate for balancing growth performance and feed efficiency in juvenile yellow perch. If maximum growth is prioritized, particularly in research or hatchery settings—feeding up to 6.2% BW/day may be appropriate, despite the reduced feed efficiency. Alternatively, 3.0% BW/day may be ideal in production scenarios where minimizing FCR is more important than achieving maximum growth.

No behavioral changes such as cannibalism or mortality were observed during the trial. Fish fed at the lowest feeding rate (1.5% BW/day) exhibited an overall weight gain of approximately 50%. The CV in body weight for the feeding treatments was 0.26, 0.18, 0.26, 0.22, and 0.24 for fish fed at 1.5%, 3.0%, 4.5%, 6.0%, and 7.5% of body weight, respectively. The CV for fish in the 1.5% group tended to be higher than that of fish fed at higher rates, indicating increased size variation. The lowest variation was observed in fish fed at 3.0%, with variation increasing as feeding rates rose. However, there was no significant correlation between feeding and CV of body size under the current study. The observed variation may be attributed to both feeding rate and sex-related growth differences. Male yellow perch typically undergo gonadal development earlier than females, which can influence growth rates. However, due to the young age of the fish in this study, sex could not be reliably distinguished.

### 4.2. Nutrient Retention and Efficiency

The patterns of nutrient retention in response to different feedings were consistent with the observed growth outcomes. At the highest feeding rate (7.5% BW/day), the highest FCR and lowest PER were found. These results suggest that while higher feeding rates can enhance growth, they reduced the efficiency with which fish convert feed protein into body mass. This inefficiency is likely due to overfeeding, which results in feed waste and higher FCR.

Analysis of energy and protein retention showed that the optimal feeding rate for maximum energy retention (ER) was higher than that for PR (3.9% BW/day vs. 1.6% BW/day). This discrepancy likely arises because protein is primarily used for growth and tissue maintenance, while energy supports a broader range of metabolic activities. Protein retention typically decreases with increasing feeding rates. Once protein requirements are met, any excess protein is converted into energy rather than being stored, which explains the decline in PR at higher feeding levels. In contrast, energy is required for maintenance, growth, and metabolism, necessitating a higher feeding rate to meet these demands. Our findings also show that the feeding rate required for maximum ER closely aligns with the feeding rate that supports optimal growth, suggesting that overall energy requirements mirror growth demands. The findings of this study also align with studies on other species, such as white sturgeon (*Acipenser transmontanus*), lake sturgeon (*Acipenser fulvescens*), cuneate drum (*Nibea miichthioides*), and Nile tilapia (*Oreochromis niloticus*), which observed reduced nutrient retention or energy retention when feeding rates exceed satiation levels [4,21,22,23]. These studies underscore the importance of carefully managing feeding rates to optimize feed utilization and minimize nutrient waste and feed cost.

### 4.3. Morphology and Body Nutrition Composition

Condition factor and HSI are commonly used as indirect indicators of fish growth, fitness, and energy storage [24,25,26]. A high CF typically reflects good nutritional status and ample energy reserves, while a low CF suggests limited access to food. The liver plays a crucial role in short-term energy storage, primarily through glycogen and fat reserves, which are replenished when nutrients are abundant. Conversely, a low HSI indicates insufficient food intake, with energy stores in the liver being depleted. This is consistent with the low CF and HSI observed in fish fed 1.5% body weight (BW)/day, which also showed reduced growth and lower energy storage. Additionally, the reduced CF and HSI values correlated with lower lipid content in these fish, further supporting the link between these indices and energy storage [4,27,28].

The results of this study demonstrate that feeding rates have varying impacts on the proximate composition of yellow perch fingerlings, depending on nutrient types. As feeding rates increased, moisture content decreased, and lipid content increased, consistent with findings from studies on species such as gilthead sea bream (*Sparus aurata*) [26], European sea bass (*Dicentrarchus labrax*) [29], and Nile tilapia [30]. Overfeeding leads to the storage of excess energy as lipids, explaining the increased fat accumulation at higher feeding rates [31]. Fish fed 1.5% BW/day exhibited higher moisture content and lower lipid levels, which corresponded with the reduced growth and energy retention observed in this group. Ash content was higher in fish fed the lower rates (1.5% and 3% BW/day), likely due to a higher proportion of bones relative to other tissues in underfed fish. Whole-body protein content remained constant across feeding treatments, suggesting that even at the lowest feeding rates, protein levels were sufficient to meet the maintenance needs of yellow perch.

### 4.4. HSP70 Expression in Tissues

The liver tissue showed a positive correlation between feeding rates and HSP70 expression, with moderate feeding rates (4.5% BW/day) promoting the highest basal expression. This underscores the role of nutrition in modulating stress response, as previously reported in previous studies [12,18,31]. Lower HSP70 expression in yellow perch under suboptimal feeding is likely due to the energy demands of stress responses, as energy is required for HSP70 synthesis. Similar reductions have been observed in other species, including white sturgeon, under low feeding or starvation [18,31], suggesting HSP70 expression is closely tied to growth status. The findings highlight the importance of higher feeding rates for supporting heat shock protein 70 expression, which indicate the capacity of stress tolerance response at the molecular level. The optimal feeding rate for peak HSP70 expression was 6.2% BW/day, while 4.6% BW/day was best for basal expression. Additionally, HSP70 expression remained elevated in fish fed 6.0% or 7.5% BW/day during the post-heat shock period, suggesting these fish may still secure energy and nutrients to sustain the HSP70 level during post-heat shock. This warrants further investigation.

In contrast to liver tissue, muscle tissue showed a less pronounced response to feeding rate changes. This may be due to the different functions involved by the liver and muscle tissues [32,33]. The liver must quickly adapt to maintain critical metabolic functions, whereas muscle primarily handles contraction and physical work, with a slower protein turnover rate. Consequently, the liver exhibits a more adaptive stress response, with a higher nutrient turnover rate compared to muscle [34,35]. Muscle serves as a long-term energy reserve, while the liver provides short-term energy supply. As a result, muscle tissue may experience slower stress response and recovery. This may explain why HSP70 levels changed less in muscle tissue than in liver tissue during acute heat shock. Additionally, HSP70 levels remained elevated in muscle during the post-heat shock period, supporting the idea that nutrient depletion occurs more rapidly in the liver than in muscle [36], leading to faster reduction of HSP70 in the liver during recovery. The response of HSP70 expression differs between liver and muscle tissues due to their distinct functions, a pattern also observed in white sturgeon and green sturgeon [37]. In addition, the absence of a control group sampled at the same time without heat shock limits the ability to directly compare heat shock versus non-heat shock conditions. This will be addressed in future studies.

The findings of this study demonstrate that HSP70 expression is closely linked to the nutritional status of yellow perch fingerlings. The upregulation of HSP70 reflects a cellular defense mechanism aimed at maintaining protein homeostasis and preventing stress-induced damage. However, relying solely on HSP70 expression to assess stress tolerance is insufficient; a comprehensive evaluation should also include physiological, behavioral, or survival metrics. This warrants further investigation in future studies. Additionally, heat tolerance in this study was assessed using an acute heat shock challenge, which may not fully capture the effects of gradual temperature changes typically experienced in yellow perch farming environments. Despite this limitation, the results offer valuable insights into the species’ capacity to cope with thermal stress. Heat shock remains a significant concern in outdoor pond systems or aquaculture operations without precise temperature regulation. As such, implementing optimal feeding strategies may serve as a proactive management approach to enhance fish resilience and stress tolerance under fluctuating thermal conditions.

## 5. Conclusions

This study demonstrates that feeding rate significantly influences growth performance, nutrient retention, and HSP70 expression in yellow perch; 4.5% BW/day is recommended as the optimal feeding rate for balancing growth performance and feed efficiency in juvenile yellow perch. If maximum growth and heat shock protein expression are the goal, feeding up to 6.2% BW/day may be appropriate, despite the reduced feed efficiency. Alternatively, 3.0% BW/day may be ideal in production scenarios where minimizing FCR is more important than achieving maximum growth. While a feeding rate of 1.5% BW/day may suffice to meet basic maintenance needs without causing weight loss or mortality, it appears to reduce the fish’s capacity for heat shock tolerance. These results suggest that HSP70 expression is closely linked to the nutritional status of fish.

Furthermore, the study reveals tissue-specific differences in HSP70 expression across feeding rates, reflecting the unique physiological roles of each tissue. Although HSP70 is a well-established biomarker for cellular stress and protection against environmental stressors, future studies should incorporate additional indicators—such as survival rates, behavioral responses, and physiological parameters—to further validate the relationship between HSP70 expression and stress tolerance. Overall, these findings provide important baseline data to guide feeding strategies and advance nutrition research in yellow perch fingerlings.

## Figures and Tables

**Table 1:** Growth performance of yellow perch fed different feeding rates for four weeks.

	Feeding Rate (% Body Weight/Day)				
Parameters	1.5	3.0	4.5	6.0	7.5
Weight gain (%)	48.3 ± 2.17 ^c^	117.1 ± 2.16 ^b^	172.8 ± 5.73 ^a^	178.5 ± 5.42 ^a^	183.8 ± 10.45 ^a^
SGR (% day^−1^)	1.41 ± 0.05 ^c^	2.77 ± 0.04 ^b^	3.58 ± 0.07 ^a^	3.66 ± 0.07 ^a^	3.72 ± 0.13 ^a^
FCR	0.82 ± 0.03 ^c^	0.75 ± 0.01 ^c^	0.83 ± 0.02 ^c^	1.07 ± 0.02 ^b^	1.33 ± 0.06 ^a^
PER (%)	2.05 ± 0.15 ^a^	2.24 ± 0.04 ^a^	2.03 ± 0.10 ^a^	1.57 ± 0.06 ^b^	1.27 ± 0.11 ^c^
PR (%)	36.3 ± 0.98 ^a^	37.0 ± 1.05 ^a^	33.6 ± 1.33 ^a^	26.2 ± 1.28 ^b^	21.4 ± 1.62 ^c^
ER (%)	31.4 ± 1.93 ^b^	44.5 ± 0.74 ^a^	40.4 ± 0.49 ^a^	31.2 ± 1.00 ^b^	26.2 ± 1.47 ^b^
CF	0.89 ± 0.02 ^b^	0.95 ± 0.01 ^ab^	1.02 ± 0.00 ^a^	1.01 ± 0.01 ^a^	1.03 ± 0.03 ^a^
HSI (%)	0.92 ± 0.07 ^c^	1.30 ± 0.10 ^ab^	1.44 ± 0.06 ^ab^	1.62 ± 0.02 ^a^	1.60 ± 0.07 ^ab^

The results are presented as means ± SE, *n* = 3. Means in the same row with different letters indicate significant differences among treatments as determined by Tukey’s HSD test (*p* < 0.05).

**Table 2:** Proximate composition of juvenile yellow perch fed different feeding rates for four weeks (% Wet Tissue).

Feeding Rate (% BW day^−1^)	Moisture	Crude Protein	Lipid	Ash
1.5	73.9 ± 0.05 ^a^	15.6 ± 0.28 ^a^	5.6 ± 0.23 ^b^	3.7 ± 0.02 ^a^
3.0	72.1 ± 0.12 ^b^	15.7 ± 0.28 ^a^	8.0 ± 0.10 ^a^	3.4 ± 0.03 ^b^
4.5	71.8 ± 0.22 ^b^	15.9 ± 0.05 ^a^	8.3 ± 0.14 ^a^	3.3 ± 0.01 ^bc^
6.0	71.6 ± 0.12 ^b^	16.0 ± 0.19 ^a^	8.2 ± 0.17 ^a^	3.2 ± 0.04 ^c^
7.5	71.3 ± 0.24 ^b^	16.1 ± 0.05 ^a^	8.7 ± 0.36 ^a^	3.2 ± 0.03 ^c^

The results are presented as means ± SE, with *n* = 3. Each replicate consists of 8 pooled fish. Means in the same column with different letters indicate significant differences among treatments as determined by Tukey’s HSD test (*p* < 0.05).

## Data Availability

The data presented in this study are available on request from the corresponding author.
